# Expectancy effects in the Autonomous Sensory Meridian Response

**DOI:** 10.7717/peerj.5229

**Published:** 2018-08-22

**Authors:** Daniella K. Cash, Laura L. Heisick, Megan H. Papesh

**Affiliations:** Department of Psychology, Louisiana State University and Agricultural and Mechanical College, Baton Rouge, LA, USA

**Keywords:** Autonomous Sensory Meridian Response (ASMR), Mindfulness, Stress management, Coping, Placebo effect

## Abstract

The Autonomous Sensory Meridian Response (ASMR) is a tingling, almost euphoric, sensation often elicited following certain visual or auditory stimulations (Barratt & Davis, 2015). Despite considerable media attention, little empirical work has investigated the underlying mechanisms. In the present study, ASMR enthusiasts and naïve observers listened to audio clips with and without ASMR-eliciting characteristics. We also manipulated participants’ expectations of ASMR, providing a measure of “placebo effects.” Although naïve participants were susceptible to suggestive instructions, experienced users were not, suggesting that initial exposure to ASMR media may evoke somatosensory responses consistent with one’s expectations. Implications for at-home stress management techniques are discussed.

## Introduction

The Autonomous Sensory Meridian Response (ASMR) is a sensory phenomenon that is typically elicited in response to visual or audio stimuli that cause static, tingling sensations that originate in the head and often spread to the neck and sometimes other regions of the body (Barratt & Davis, 2015). In addition to these tingles, ASMR users often report feeling more relaxed and content following the ASMR experience, leading some to suggest that ASMR can be used as a treatment for stress, depression, chronic pain, and as an at-home relaxation tool (Taylor, 2013), although its users vary in their reported intent when engaging with ASMR materials (Marsden, 2012; see also ASMR University, 2018). ASMR is similar to other sensory phenomena, such as *frisson*, the tingling sensations that are often associated with emotional responses to music (e.g., “chills;” Del Campo & Kehle, 2016), and *synesthesia*, a neurological condition in which individuals experience multiple senses concurrently (e.g., visually presented material may be associated with specific scents). Despite these similarities, ASMR is associated with several physiological experiences that set it apart from other sensory phenomena. For example, although frisson and ASMR can both be described as “pleasant tingling,” their time courses differ: frisson typically occurs and spreads quickly, while ASMR has idiosyncratic durations, coupled with sensations that often spread to other bodily areas with increasing intensity (Barratt & Davis, 2015). Similarly, although ASMR and synesthesia are both produced consistently to specific stimuli, individuals can choose to disengage from ASMR, while synesthetic experiences are uncontrollable (Hubbard & Ramachandran, 2005; Lupiáñez & Callejas, 2006).

ASMR has enjoyed considerable media attention, having been hailed as a “must try” remedy for the stressors of daily life (Gibson, 2014). Despite its current popularity, ASMR was unknown until the late 2000s (ASMR University, 2018; Del Campo & Kehle, 2016), when users on an Internet forum (Reddit) began questioning whether other individuals felt the same tingling sensations while watching various YouTube videos. In 2010, these discussions led to the phenomenon being colloquially termed the ASMR, with each word of the name describing a component of ASMR sensations: the feeling is *autonomous*, in that individuals are assumed to have no control over initiating it, and *sensory*, in that it is a physical response that occurs along the body’s *meridian*, or center (Young & Blansert, 2015). Having a name for the experience resulted in a surge of interest in both the experiences and characteristics of stimuli that elicit ASMR. This curiosity shows no signs of abating, as there now exist many forums and websites dedicated to ASMR, including an ASMR-specific Reddit forum (www.reddit.com/r/ASMR) with over 131,735 subscribers. Moreover, when ASMR is featured in popular media, it is often described using attention-grabbing terms, such as “brain orgasms” and “whisper porn” (Beck, 2013; Milzoff, 2015), which serve to expose more people to the potential benefits of ASMR but may also contribute to significant confusion about what the phenomenon entails.

Despite popular interest in ASMR, relatively little research has examined the phenomenon, and very few studies have been empirical. In one of the first assessments of ASMR, Barratt & Davis (2015) categorized common triggers for ASMR, and where these sensations were often experienced on the body. They found that the top triggers for ASMR included audio or visual stimuli that depicted whispering, personal attention, crisp sounds (e.g., metallic foil, tapping fingernails), and slow or repetitive movements. When presented with such stimuli, participants often reported the characteristic tingling sensations as originating at the top of the head, and then traveling down the spine toward the rest of the body. Moreover, Barratt & Davis (2015) found that these ASMR experiences were often associated with self-reported temporary reductions in chronic pain, and/or improvements in mood, a finding that resembles the benefits demonstrated with mindfulness-based stress reduction (MBSR) therapy and yoga.

Although Barratt & Davis (2015) surveyed individuals who reported regularly watching ASMR media and experiencing ASMR, participants’ self-reports differed in common triggers, physical experiences, and psychological outcomes. For example, whereas 38 participants reported that ASMR improved their chronic pain, 40 reported that ASMR had no effect on their pain symptoms. These, and other, differences suggest the existence of individual differences in ASMR susceptibility and consequences. Fredborg, Clark & Smith (2017) used the Big Five Personality Inventory to investigate the personality traits that are associated with individuals who experience ASMR, vs. those who do not. Relative to individuals who did not report experiencing ASMR, those who did were found to have higher scores on the Openness-to-Experience and Neuroticism measures, but lower scores on Conscientiousness, Extraversion, and Agreeableness. Fredborg, Clark & Smith (2017) suggested that the differences between groups on the Openness-to-Experience measure are related to ASMR users’ heightened sensitivity to aesthetic and sensory experiences. Similarly, McCrae (2007) found that individuals who experienced frisson were likely to score high on Openness-to-Experience, suggesting additional similarities between frisson and ASMR. Further, although Neuroticism is often associated with “negative” traits (e.g., anger, hostility, anxiety; John & Srivastava, 1999), Fredborg, Clark & Smith (2017) suggested that ASMR users scored higher on this measure due to frequent concurrent reports of depression (Barratt & Davis, 2015), although it is important to note that susceptibility to ASMR experiences has not been linked with predisposition for depression. That said, one of the therapeutic uses of ASMR is the temporary relief of depression and stress.

In addition to personality characteristics, there exist individual differences in functional neural connectivity across individuals who do and do not experience ASMR. Smith, Katherine Fredborg & Kornelsen (2017) examined activity in the default mode network (DMN) in 11 individuals who experienced ASMR compared to 11 who did not. The DMN consists of a distributed network of interconnected brain regions (see Buckner, Andrews-Hanna & Schacter, 2008; Raichle, 2015) in which neural activity covaries in the absence of internal or external stimulation (hence the name “default”). Using resting-state fMRI, Smith, Katherine Fredborg & Kornelsen (2017) found that individuals who reported experiencing ASMR showed reduced functional connectivity, relative to individuals who did not report experiencing ASMR. This reduced functional connectivity was hypothesized to reflect the inability to inhibit sensory-emotional experiences, as similar changes in functional connectivity are observed in disorders with sensory-emotional deficiencies (e.g., autism, Kennedy & Courchesne, 2008; schizophrenia, Bluhm et al., 2007). In addition, individuals who reported experiencing ASMR show greater functional connectivity between regions not typically considered part of the DMN (e.g., occipital, frontal, and temporal cortices), suggesting that ASMR may be linked to the recruitment or involvement of several resting-state networks.

Given the potential health benefits of using ASMR for stress reduction, more research is needed to better understand the underlying psychological and neurological mechanisms. Among many unanswered questions about the psychology behind ASMR is whether the phenomenon truly exists, or rather is a product of individual expectations. Although many people report using the phenomenon as a stress reduction technique, many other individuals report not experiencing ASMR at all (e.g., control participants in empirical ASMR investigations; Fredborg, Clark & Smith, 2017; Smith, Katherine Fredborg & Kornelsen, 2017). Moreover, does the veracity of the effect matter? For example, perhaps ASMR users are experiencing placebo effects, stress reduction because they *expect* to experience stress reduction. Such a placebo effect is not without precedent. For example, Greenwald et al. (1991) observed improvements in participants’ memory and self-esteem when they were given audiotapes merely labeled as containing subliminal messages geared toward improving those domains. Although the experience was a placebo effect, it was nevertheless beneficial. Moreover, an important consideration in measuring the efficacy of any medical or psychological intervention is the role of expectations (Boot et al., 2013), which can powerfully sway individuals’ subjective experiences. Indeed, Boot et al. (2013) suggest that, without measures of expectations, claims about the efficacy of interventions should be considered with caution, or not at all.

Regardless of whether ASMR is a real and/or placebo effect, it potentially carries important implications for at-home stress management programs. As a first step toward better understanding ASMR, the goal of the current study was to assess whether ASMR is affected by individuals’ expectations or if the phenomenon emerges regardless of expectations. To address this question, we presented ASMR users and naïve participants with audio clips that were and were not expected to produce ASMR. Additionally, we varied whether participants were told to expect the ASMR experience for the clips, allowing us to better appreciate the role of expectations in ASMR. To preface the results, we found that ASMR users were immune to our expectation manipulation, but naïve users experienced ASMR when they were told to expect it and did not experience ASMR when told not to expect it. These results have implications for at-home stress management programs, and we discuss the benefits of placebo effects in the “General Discussion.”

## Method

### Participants

A total of 209 volunteers completed the experiment. Of these, 107 were college students (*M*_age_ = 19.64, SE = 1.42; 80 female), recruited from Psychology courses, who received partial course credit in exchange for participating. The remaining 102 participants (*M*_age_ = 26.76, SE = 8.06; 32 female) were recruited from an Internet forum dedicated to the ASMR phenomenon (https://www.reddit.com/r/asmr/). These participants volunteered their time and were not compensated. Participants were randomly assigned to Instruction conditions (described below), such that 103 received encouraging instructions and 106 received discouraging instructions (see Table 1 for a full demographic breakdown^1^1Note that, like studies examining the benefits of doing yoga (e.g., Granath et al., 2006; Sharma, 2014), the majority of ASMR users in our sample were White.). This research was conducted with Institutional Review Board approval granted by Louisiana State University (approval E10038). All participants provided informed consent prior to beginning the study.

**Table 1:** Participant demographics.

Group	Encouraging Instructions					
	*Asian*	*Black*	*Hispanic*	*White*	*Other*	*Total*
Naïve	3	11	1	38		53
Reddit	1		2	46	1	50

Group	Discouraging Instructions					
	*Asian*	*Black*	*Hispanic*	*White*	*Other*	*Total*
Naïve	2	11	3	38		54
Reddit	4		2	44	2	52

### Materials

Barratt & Davis (2015) identified whispering, personal attention, crisp sounds, slow movements, and repetitive movements as the top five most popular ASMR triggers. Using ASMR-specific YouTube videos, we converted whispering, personal attention, and crisp sounds (specifically, finger tapping) into 5-min audio files (see Table 2 for descriptions and links). Because we could not identify auditory correlates for slow movements and repetitive movements, we replaced these categories with 5-min audio clips of white noise and repetitive noises (specifically page turning), both of which were indicated for ASMR use. As summarized in Table 2, we also gathered matched “foils” for each of the ASMR clips. These alternative clips were selected to maintain passing similarity to the matched ASMR clips, but contained elements that often preclude the ASMR phenomena (e.g., lack of repetition, unanticipated breaks in the audio presentation). Lastly, we included a “control” clip, instrumental technological music, that has not been previously associated with ASMR, but rather with frisson. Indeed, many of the Reddit users commented on how odd it was for us to include this clip in an ASMR study, and provided feedback outlining the differences between ASMR and frisson in the post-experiment questionnaire (see Appendix). Although recent work has shown the interplay between visual and auditory stimulation may play a role in ASMR experiences (Barratt, Spence & Davis, 2017), auditory stimuli were used in order to maintain experimental control, and to avoid potential confounds from creating foil stimuli that too closely (or too distantly) resembled ASMR material.

**Table 2:** Stimuli, and links to original videos.

Category		
*Clip Type*	Description	Link
Personal Attention		
*ASMR*	A roleplay clip of an esthetician visit	https://www.youtube.com/watch?v=LMs-VIbc_4
*Foil*	A clip of someone narrating his life experience	https://www.youtube.com/watch?v=sl_f9tmAP64
Whispering		
*ASMR*	A clip of a female whispering	https://www.youtube.com/watch?v=RVpfHgC3ye0
*Foil*	A clip of a female screaming	https://www.youtube.com/watch?v=GPJ1uQwmNHk
Crisp Sounds		
*ASMR*	A clip of someone tapping	https://www.youtube.com/watch?v=jbvxlvvrLuU
*Foil*	A clip of someone finger drumming	https://www.youtube.com/watch?v=HjC-hPvihHI
Repetitive Noise		
*ASMR*	A clip of someone turning pages	https://www.youtube.com/watch?v=N7jwQi2e4rY
*Foil*	A clip of someone playing piano scales	https://www.youtube.com/watch?v=fCxmDUl8__U
White Noise		
*ASMR*	A clip of nonstop hair dryer noise	https://www.youtube.com/watch?v=eJT8xuI_5PY
*Foil*	A clip of one half of a phone conversation	https://www.youtube.com/watch?v=TwPvphTxoG8
Control*		
*All*	An excerpt from a technological music song	https://www.youtube.com/watch?v=iXAbte4QXKs

**Note:**

* The Control clip was the same for each group.

### Procedure

After providing informed consent, participants received written instructions. All participants read the following:

The Autonomous Sensory Meridian Response (ASMR) is a sensory phenomenon in which people experience a pleasant tingling, static-like sensation in response to listening to specific audio or visual stimuli.

Those in the encouraging instructions condition then read:

Over the course of this experiment, you will hear audio from 3 different video presentations. All of the audio files that you are about to hear have been shown to produce the ASMR effect, and we are interested in the causal mechanism causing the response. Please listen to the clips, because you will be asked to rate the degree to which you experienced the tingling, static sensation of the ASMR.

Those in the discouraging instructions condition instead read:

On the following pages, you will hear 3 different audio clips. None of the clips that you are about to hear have been shown to produce the ASMR effect, and we are interested in determining which audio-visual characteristics prevent ASMR effects from occurring. Please listen to the clips, because you will be asked to rate the degree to which (if at all) you experienced the tingling, static sensation of the ASMR.

Prior to playing the first clip, participants read that they would listen to each clip for 5 minutes, before rating their ASMR experience on a Likert scale from one (none at all) to seven (a great deal). Participants then indicated whether they understood the instructions and wished to proceed.

In addition to randomly assigning participants to Instruction conditions, they were also randomly assigned to one of the five ASMR category conditions. Although participants would have ideally listened to all categories, we manipulated this between-subjects to keep the experiment a reasonable length, as each category contained three 5-minute clips (see Table 2), and participants’ ASMR ratings were not queried until the end of each clip. After providing responses for all three clips, participants filled out a demographic questionnaire and provided answers to follow up questions pertaining to their experience with ASMR, health, and whether they were able to discern the nature of the study (see Appendix). Upon completion, participants were thanked and debriefed.

## Results

Prior to analyzing the data, we ensured that the student sample was naïve (i.e., no prior experience with ASMR videos) and the Reddit sample was familiar with ASMR by examining their post-experimental answers about previous experience with the phenomenon. Whereas none of the student sample reported having watched ASMR videos, 99 participants from the Reddit sample reported regular viewing of ASMR media (the three Reddit participants who indicated not watching ASMR videos seem to have selected “no” in error; each of these participants subsequently described their ASMR viewing habits and triggers). The Reddit participants’ ASMR sensations are summarized in Table 3, and 92.3% of these participants identified their head as the origination point for their experience (or as one of several origination points). The subjective sensory and origination point data are consistent with prior literature on ASMR (Barratt & Davis, 2015). Please see Table 4 for means and standard deviations for ratings across all conditions.

**Table 3:** Percentage of ASMR viewers triggered by different video types.

Trigger	Percentage of participants (%)
Personal attention	75.70
Whispering	65.00
Crisp sounds	64.00
Slow movements	47.60
Repetitive tasks	31.10
Water pouring	21.40
Smiling	9.70
Laughing	2
Vacuuming	1
Aeroplane noises	0.00

**Table 4:** Means and standard deviations for participants ASMR ratings.

		Instruction type	
		Encouraging	Discouraging
Naïve participants	ASMR	3.45 (2.00)	2.52 (1.85)
	Foil	3.13 (1.78)	1.96 (1.40)
	Control	3.53 (2.02)	2.26 (1.46)
ASMR participants	ASMR	3.50 (1.71)	3.38 (2.05)
	Foil	1.68 (1.28)	1.40 (1.05)
	Control	1.66 (.94)	1.27 (.69)

Because we were interested in whether participants’ expectations played a role in their experience with ASMR and ASMR-like (which would not be expected to induce ASMR) videos, we analyzed the data with and without 12 participants who either guessed the study hypothesis in the post-experiment questionnaire, or revealed a familiarity with more than one of the video clips (11 of these participants were from the Reddit group). The patterns in the data were the same regardless of inclusion, so we present the full analyses. Degrees of freedom are Greenhouse-Geisser corrected, when needed.

**Figure 1:**
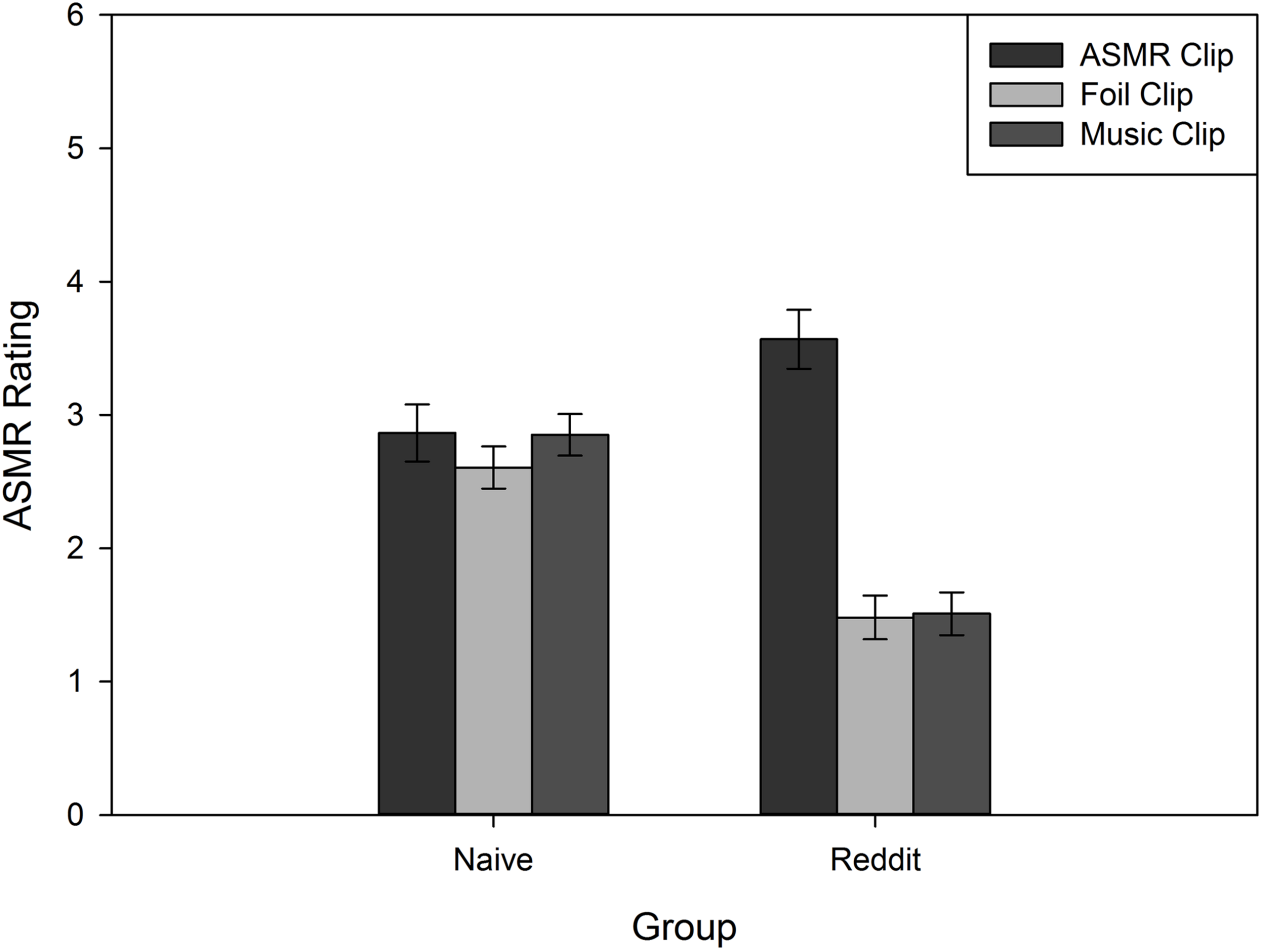
Average ASMR rating for Naïve and Reddit participants after listening to ASMR, Foil, or Music clips. Error bars represent ±1 SEM.

**Figure 2:**
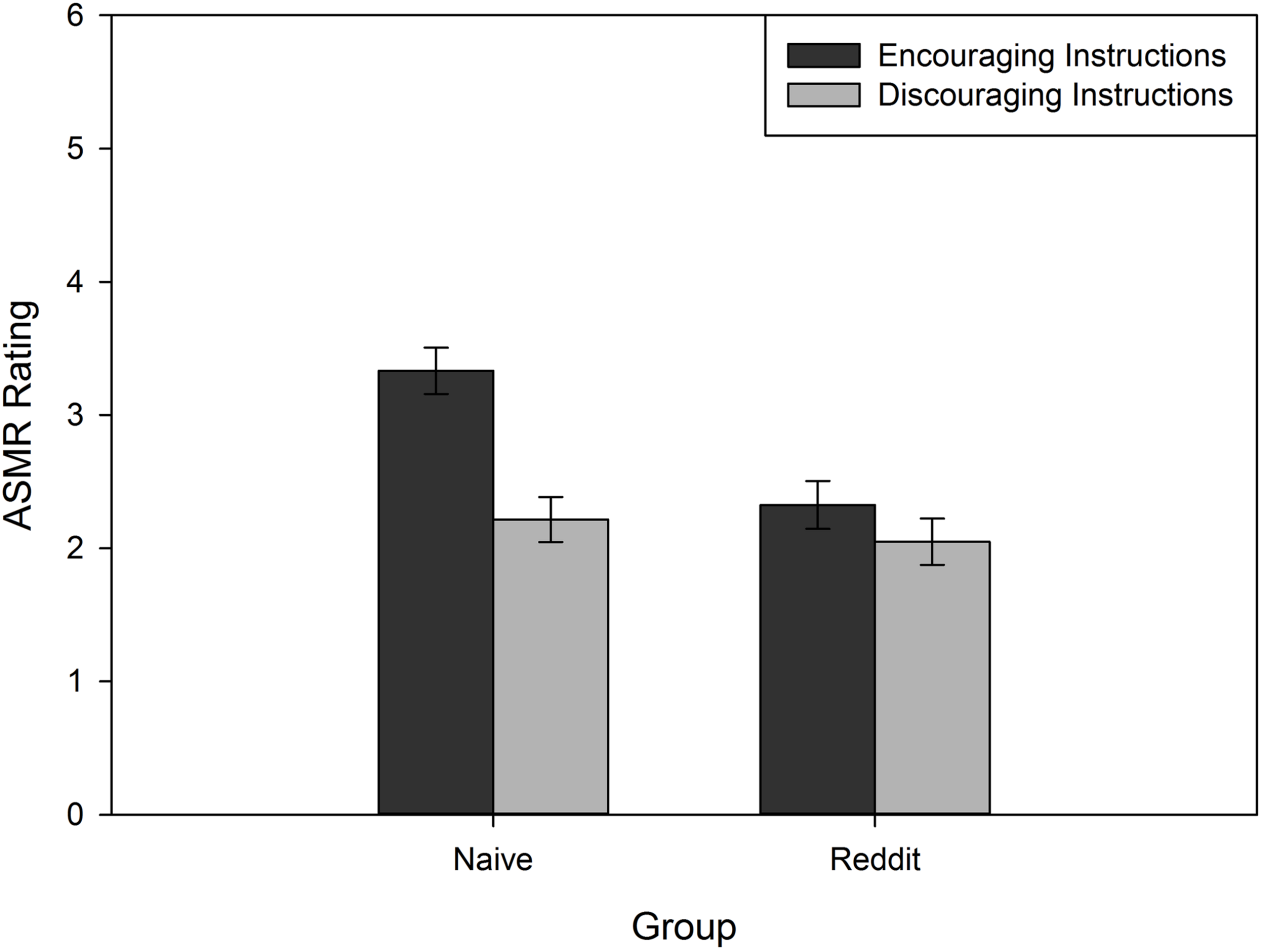
Average ASMR rating for Naïve and Reddit participants who received Encouraging or Discouraging instructions. Error bars represent ±1 SEM.

To determine whether ASMR effects can be elicited by suggestion, and whether this differs based on existing ASMR experience, we analyzed participants’ post-video ratings of their “ASMR tingling, static-like sensations” in a 2 (Instruction: Encouraging, Discouraging) × 3 (Clip Type: ASMR, Foil, Music) × 2 (Group: Naïve, Reddit) mixed model ANCOVA, with Instruction and Group serving as between subjects measures, and Age and Gender included as covariates. Although there was a main effect of Clip Type, *F*(1.94, 393.6) = 5.16, *p* = 0.007, η_p_^2^ = 0.03, it was qualified by an interaction with Group, *F*(1.94, 393.6) = 21.69, *p* < 0.05, η_p_^2^ = 0.10. As shown in Fig. 1, this interaction was driven by the Reddit participants, who experienced stronger ASMR sensations to the ASMR clips, relative to the foil and music clips. No such differences were observed in the Naïve group. Moreover, the Reddit group had significantly lower ASMR ratings for the foil and music clips, relative to the Naïve group. To fully explore the patterns observed in Fig. 1, we conducted analyses to determine if ratings differed between groups for the Clip Types based on Instruction. We conducted independent samples *t*-tests comparing these ratings. With the exception of participants in the encouraging instructions condition who were rating the ASMR clips (*t*(101) = −0.13, *p* = 0.90), all other differences in ratings were significant. For participants in the encouraging condition, naïve participants were significantly more likely to provide higher ratings for both the foil, *t*(94.74) = 4.77, *p* < 0.001, *d* = 0.94, and music clips, *t*(74.32) = 6.06, *p* < 0.001, *d* = 1.18, than the Reddit group. With regard to the discouraging instructions, the Reddit group provided higher ratings to the ASMR clips than the naïve group, *t*(104) = −2.29, *p* = 0.02, *d* = 0.44. However, for the foil, *t*(98.29) = 2.33, *p* = 0.02, *d* = 0.45, and music, *t*(76.25) = 4.5, *p* < 0.001, *d* = 0.87, clips, Reddit participants provided significantly lower ratings, relative to the naïve group. Similarly, the main effects of Instruction, *F*(1, 203) = 18.89, *p* < 0.001, η_p_^2^ = 0.09, and Group, *F*(1, 203) = 7.93, *p* = 0.005, η_p_^2^ = 0.04, reliably interacted, *F*(1, 203) = 6.82, *p* = 0.01, η_p_^2^ = 0.03. As shown in Fig. 2, the encouraging instructions only affected ASMR ratings in the Naïve group. Participants from the Reddit group were not affected by suggestive instructions.

**Figure 3:**
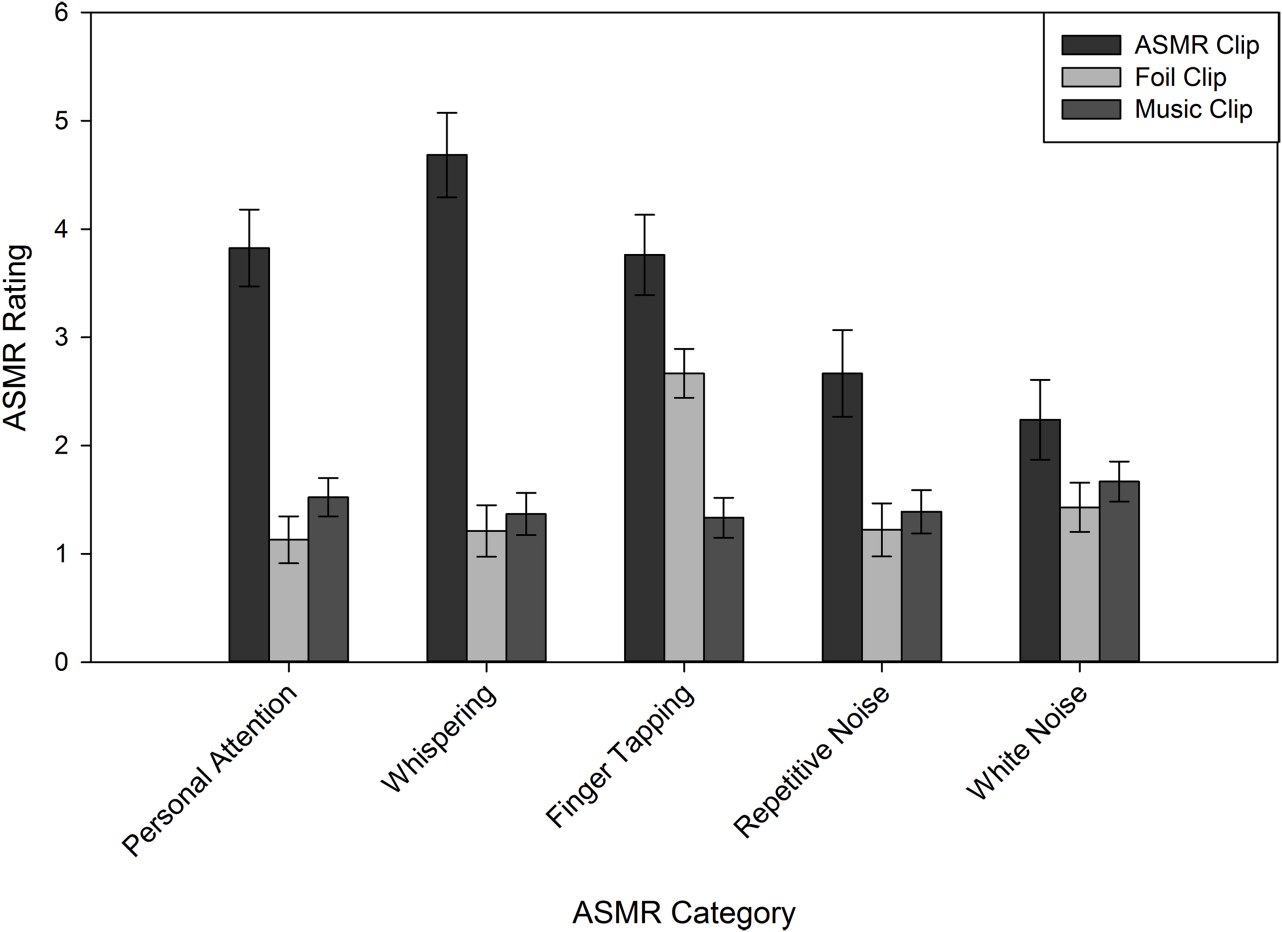
Average ASMR rating for Reddit participants, following each clip type. Error bars represent ±1 SEM.

**Figure 4:**
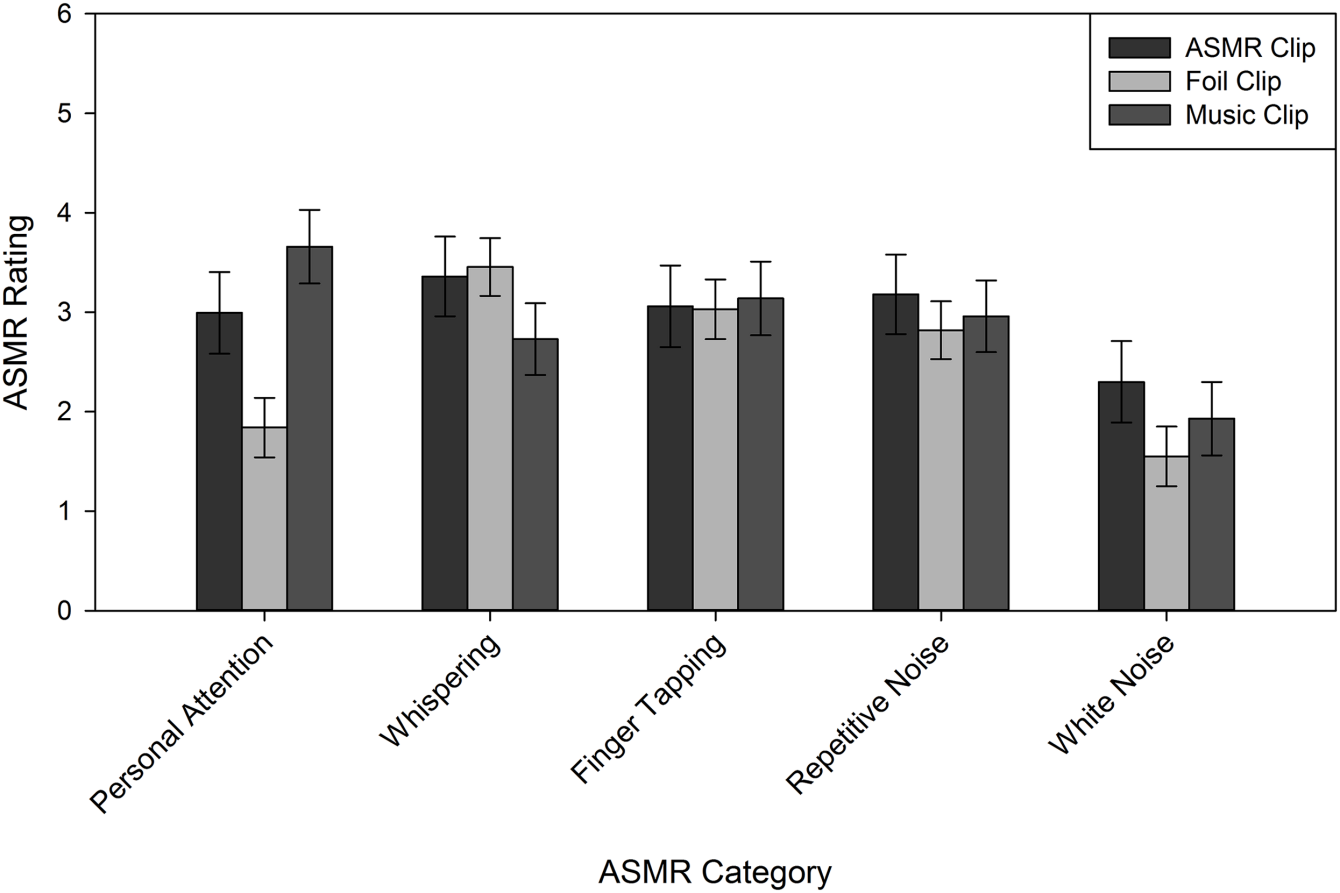
Average ASMR rating for Naïve participants, following each clip type. Error bars represent ±1 SEM.

Because the Reddit group was not affected by instruction manipulations, we next sought to determine whether they were affected more/less by specific types of ASMR videos, as suggested by their triggers in Table 2. To this end, we analyzed their ASMR ratings in a 3 (Clip Type) × 5 (Category: personal attention, tapping, repetitive noise, white noise, and whispering) mixed model ANOVA, with Category as the between-subjects variable. Both main effects (Clip Type: *F*(1.68, 163.3) = 100.6, *p* < 0.001, η_p_^2^ = 0.51; Category: *F*(4, 97) = 3.84, *p* = 0.006, η_p_^2^ = 0.14) were qualified by an interaction, *F*(6.74, 163.3) = 7.81, *p* < 0.001, η_p_^2^ = 0.24. As shown in Fig. 3, for every category except white noise, the ASMR clip yielded the highest ASMR ratings, relative to foil and music clips. We performed a similar analysis on the Naïve group, including the Instruction factor because of the omnibus results. This analysis also yielded a Clip Type by Category interaction, *F*(7.88, 190.9) = 2.77, *p* = 0.007, η_p_^2^ = 0.10. Unlike the Reddit group, only the personal attention clips yielded reliable differences: both the ASMR (*M* = 2.99, SE = 0.41) and music (*M* = 3.66, SE = 0.37) clips produced higher ASMR ratings, relative to the foil clips (*M* = 1.84, SE = 0.30; see Fig. 4). The ASMR and music clips did not reliably differ.

## General Discussion

In this study, we examined the role of expectations in eliciting a sensory phenomenon known as ASMR, which is said to manifest as a pleasant tingling sensation, followed by improved mood and/or stress (Marsden, 2012; Taylor, 2013; Barratt & Davis, 2015). Given the suggested benefits of ASMR, and the ease of watching ASMR media in one’s own home, we sought to determine whether these subjective sensory experiences may represent a placebo effect. To this end, both naïve observers (i.e., individuals who were unfamiliar with ASMR), and those who reported regularly viewing ASMR media (i.e., participants from a Reddit forum dedicated to the phenomenon), read suggestive instructions prior to listening to three audio clips. Each participant listened to one clip that was selected from a pool of media intended for ASMR use, one that merely resembled popular ASMR categories, and one clip that was not similar to any standard ASMR media. We observed that naïve participants reported experiencing ASMR when the audio clips they heard were prefaced by leading instructions, while experienced ASMR users only reported experiencing ASMR when the audio clips were those intended for ASMR use.

The role of expectations can be powerful in psychology and pain/stress management. For example, when participants are lead to believe that subliminal self-help tapes will improve their self-esteem, they report having improved self-esteem, even if the tapes contained no such content (Greenwald et al., 1991). The placebo effect is well-documented, even in clinical contexts (Finniss et al., 2010; Pollo et al., 2001; Colloca & Benedetti, 2005; Price, Finniss & Benedetti, 2008; Kaptchuk & Miller, 2015), and provides users with a sense of improvement or relief in an expected direction and domain. These improvements occur regardless of actual pharmaceutical or psychological intervention, but are nevertheless experienced as “real” changes. Because ASMR users often report using the experience to relieve symptoms of depression or chronic pain (Barratt & Davis, 2015), the consequences of ASMR may represent a placebo effect: Users expect reductions in their symptoms, so they experience reductions in their symptoms. Although temporary placebo-induced symptom reductions are certainly beneficial, they may prevent users from seeking more lasting medical or psychological intervention.

As with pharmaceutical placebo effects (Pollo et al., 2001), naïve participants who were led to believe that media clips would produce ASMR effects reported experiencing those effects, even when the clips were selected from sources lacking the characteristics of typical ASMR media. This expectancy effect is an important first step in understanding the utility of ASMR for stress management, as many people report improved mood and decreased stress following ASMR sensations (Marsden, 2012; Taylor, 2013; Barratt & Davis, 2015). Even if the primary sensory experience results from expectancy effects, users may nevertheless experience psychological benefits. The extent and duration of these improvements should be the target of future research on ASMR outcomes.

Although experienced ASMR participants were unaffected by the expectation manipulation (via the suggestive instructions), their ASMR ratings indicate that they were affected by their own expectations, driven by their history of ASMR viewing or participation in ASMR discussion boards. As shown in Fig. 1, the Reddit sample experienced the greatest ASMR following the ASMR clips. Their self-reports following the foil and music clips, however, fall *below* those from naïve participants, suggesting that ASMR users either recognized the ASMR clips, or were familiar enough with the characteristics of “real” ASMR media to report effects consistent with their expectations (i.e., intentionally reporting lower ASMR ratings). Although we did not predict expectancy effects from this source, the finding is nevertheless consistent with an explanation of ASMR based on individuals’ expectations.

Overall, our results suggest that ASMR may be explained by expectations, and any stress reduction or improved psychological function resulting from ASMR may be placebo effects. This, however, does not diminish the utility of ASMR for those who report experiencing it. Given the similar outcomes produced by both mindfulness exercises and the ASMR experience, part of ASMR’s utility may lie in its resemblance to guided meditation (Young & Blansert, 2015) or mindfulness practices (Chiesa & Serretti, 2009; Khoury et al., 2015; Del Campo & Kehle, 2016). The health benefits of guided meditation and ASMR are similar, and include insomnia relief, stress reduction, and relaxation (Chiesa & Serretti, 2009). Additionally, there is evidence suggesting that mindfulness meditation and placebo effects rely on similar brain areas (e.g., cortical areas responsible for generating and maintaining cognitive expectations, such as the prefrontal cortex and the anterior cingulate cortex), and that both rely on cognitive regulation of emotional experiences (Chiesa, Brambilla & Serretti, 2010). Our results are consistent with these findings, and suggest that expectations may play a role in whether and how individuals experience ASMR.

While our findings support expectancy effects from both experienced ASMR users and naïve participants, we note that our research has several limitations. First, these results do not explicitly rule out the possibility that there are preexisting physiological differences between the two groups that contribute to susceptibility to ASMR experiences. Despite the limited experimental literature investigating ASMR, there is evidence that individuals who experience ASMR may have reduced functional connectivity between the frontal lobes and sensory brain regions (e.g., the parietal cortex), as well as decreased DMN connectivity (Smith, Katherine Fredborg & Kornelsen, 2017). These differences may reflect underlying biological differences between those who experience ASMR, reflecting blending of sensory information or inability to inhibit sensory-emotional experiences, relative to those who do not. Our results cannot speak to this potential, but the behavioral data are consistent in that those who consistently seek out ASMR respond only to content intended to induce the experience, while those who do not respond consistent with suggestive instructions.

Second, despite our best efforts to recruit participants who were unfamiliar with ASMR material, we cannot guarantee that the participants in our study were truly unfamiliar, had never (or even were able to) experience ASMR, and that they did not regularly seek out ASMR content beyond the bounds of our experiment. Instead, we were limited to self-reported items that relied on their response indicating that they were unfamiliar with the phenomenon. This constitutes a limitation to our design, but does not negate our findings: even if participants were familiar with ASMR content but indicated they were not, naïve participants’ data support the conclusion that expectancy effects contribute to experiences consistent with ASMR. Finally, our results are limited by the possibility that naïve observers may have experienced *frisson*, and not ASMR effects. If ASMR experiences are indeed based in underlying physiology, it is possible that the naïve participants in our study were incapable of experiencing ASMR at all and instead mistook any pleasant tingling or static-like sensations as the relatively more common experience of frisson (see Del Campo & Kehle, 2016; Colver & El-Alayli, 2016). We think this conclusion is unlikely for two reasons. First, because there are no currently documented underlying biological differences between ASMR users and non-users, we do not think it best explains the consistency of our findings. Second, experiences of frisson are most closely linked to music (Goldstein, 1980). Only one of the clips in our experiment consisted of music, which makes it unlikely that *all* of the clips used, regardless of their consistency with ASMR media, produced frisson (see also Barratt, Spence & Davis, 2017). Future work is needed to fully differentiate the causal mechanisms of ASMR, and how underlying neural activity may contribute to ASMR experiences.

Although ASMR effects may represent placebo effects resulting from individuals’ expectations, they remain useful for many people. In recent years, relaxation (or “mindfulness”) techniques have been gaining popularity (Valley Health System, 2017). Major companies (e.g., Google, Target, others) have even adopted mindfulness training, and encourage employees to engage in these techniques during the workday. Proponents of mindful relaxation techniques often point to health benefits associated with decreasing stress, including improvements in pain management, mental health, and sleep (Dahlgren, Kecklund & Åkerstedt, 2005; Staufenbiel et al., 2013; Vachon-Presseau et al., 2013). For example, MBSR is a formalized meditation technique originally developed to aid patients suffering from chronic pain (Kabat-Zinn, 1982; Kabat-Zinn, Lipworth & Burney, 1985; Kabat-Zinn et al., 1987). This technique focuses on enhancing patients’ awareness of moment-by-moment experiences by directing attention to bodily sensations (Chiesa & Serretti, 2009). These techniques have proven beneficial for clinical and non-clinical populations suffering from physical, psychosomatic, and psychological disorders (Grossman et al., 2004; Ledesma & Kumano, 2009; Chiesa & Serretti, 2009; Bohlmeijer et al., 2010; Khoury et al., 2015; Zainal, Booth & Huppert, 2013). Taken together, these studies suggest that participants can heighten their awareness of bodily sensations, which could lead to improved physical and mental well-being.

Given the physical and psychological consequences of chronic stress (e.g., immune system suppression, rumination, gastrointestinal discomfort, reduced energy; Lupien et al., 2009), programs that provide individuals with tools to reduce stress are increasingly important. Although MBSR is typically conducted in group settings, there also exists a need for methods that can be applied from the home. Some of the benefits of ASMR over other stress reduction techniques include their cost efficiency and ease of use. ASMR videos are freely available online, and can be viewed in one’s own home, without requiring group sessions or gym memberships. Unlike other at-home stress relief practices (e.g., yoga; Granath et al., 2006; Sharma, 2014), there are no physical requirements of ASMR, aside from the ability to hear. Further, there exist many online resources (e.g., YouTube videos, popular media articles) and forum-based communities (e.g., Reddit, ASMR Lab) for and about individuals interested in ASMR, making the topic highly approachable and easily accessible.

## Conclusions

Despite its large user base and increasing popularity, we still do not precisely understand ASMR’s causal mechanisms, or why some individuals experience the phenomenon while others do not. Our results contribute to the growing literature on ASMR by suggesting that, at least for some people, ASMR may result from expectancy effects: observers who believe that certain media will result in sensory experiences will have those sensory experiences. It remains to be determined, however, whether these expectations can also influence the psychological outcomes often attributed to ASMR. Specifically, will people experience placebo stress relief, following suggestive induction of ASMR? The answer to this question has important implications for at-home stress and pain management programs, and should be the focus of new research.

## Appendix

*Follow-up questions*.

1. Do you suffer from any chronic pain or illness? If no, please write N/A. If yes, please describe below.
2. Synaesthesia is defined as perception in one sense triggering sensation in another, unstimulated sense. For example, you may “see” the letters as having colors, or sense shapes from music. Do you have any type of Synesthesia? If no, please write N/A. If yes, please describe.
3. Do you take any medications? If no, please write N/A. If yes, please describe.
4. Do you watch ASMR videos normally?
5. If you answered yes to the previous question, how many ASMR videos do you typically watch in a single session? Please enter N/A if this does not apply to you.
6. If you answered yes, please indicate what time of day you usually watch ASMR videos.
  - Upon waking
  - Mid-morning
  - Mid-day
  - Afternoon
  - Evening
  - Before sleeping
  - Whenever I have time
  - Does not apply because I do not watch them
7. If you experience ASMR, do you require specific conditions (e.g., busy room, bright lighting, etc.) to achieve it? If yes, please describe. If no please write N/A.
8. Do you feel a tingling sensation when watching ASMR videos?
9. Are these tingling sounds triggered by specific stimuli?
10. If you answered yes to the previous question, please select the items that trigger your tingling sensations while viewing ASMR videos. If you answered no, please select the N/A option.
  - Crisp sounds (e.g., tapping, crinkling plastic)
  - Whispering
  - Water pouring
  - Personal attention (e.g., face touching)
  - Vacuuming
  - Airplane noise
  - Laughing a lot and doing things that make you happy
  - Smiling
  - Watching repetitive tasks (e.g., towel folding)
  - Slow movements
  - Not applicable to me
11. If you have any other triggers, please list them below. If this does not apply, please write N/A.
12. Do any stimuli stop or prevent this tingling sensation from continuing? If yes, please describe. If no, please write N/A.
13. Please indicate where your tingles originate.
  - Head
  - Shoulders
  - Chest
  - Back
  - Arms
  - Stomach/lower abdomen
  - Genitals
  - Hips
  - Legs
  - Feet
  - This does not apply to me because I do not experience ASMR
14. Prior to this study, had you heard any of the clips that we used in the study? If you have heard one or more of the clips that were used, please write which one(s) in the box below. If you did not hear any of the clips previously, please write “N/A.”
15. What do you think the purpose of this study was?

## Supplemental Information

10.7717/peerj.5229/supp-1 Supplemental Information 1 Full data set.

## Additional Information and Declarations

### Data Availability

The following information was supplied regarding data availability: The raw data is available in a Supplemental File.
